# Protocol for a systematic review and meta-analysis on the effect of hippotherapy and related equine-assisted therapies on motor capabilities in children with cerebral palsy

**DOI:** 10.1186/s13643-020-01297-7

**Published:** 2020-03-05

**Authors:** Martin Häusler, Nicole Heussen

**Affiliations:** 1 Division of Neuropediatrics and Social Pediatrics, Dept. of Pediatrics, University Hospital RWTH Aachen, Pauwelsstrasse 30, Aachen, 52074 Germany; 2 Department of Medical Statistics, RWTH Aachen University, Pauwelsstrasse 19, Aachen, 52074 Germany; 3 Center of Biostatistics and Epidemiology, Medical School, Sigmund Freud University, Freudplatz 1, Vienna, 1020 Austria

**Keywords:** Cerebral palsy, Hippotheraphy, Equine-assisted therapy, Motor capability, GMFM, Quality of life

## Abstract

**Background:**

Equine-assisted treatments of the motor system appear to have an effect on the neuromuscular system and aim to improve the pathological condition of children with cerebral palsy. Hippotherapy is a distinct form of equine-assisted therapy where certified physiotherapists use the horse as a dynamic tool in a medical treatment setting. The objective of the proposed review is to summarize and critically appraise the evidence on the effect of equine-assisted treatments on motor capabilities of children with cerebral palsy.

**Methods:**

We will identify trials through systematic searches of PubMed, Embase, Web of Science, and the Cochrane Central Register of Controlled Trials (CENTRAL). Quality assessment of retrieved articles will be conducted using the criteria outlined in the revised tool to assess risk of bias in randomized trials (RoB 2.0) or the ROBINS-I tool (Risk Of Bias In Non-randomized Studies - of interventions), respectively. Quantitative data synthesis will be performed if treatments, participants, and the underlying clinical question are homogenous and provide adequate outcome data for meta-analysis. Otherwise, data will be synthesized, using the narrative synthesis approach.

**Conclusion:**

This review will provide a critical summary of the evidence regarding the impact of equine-assisted treatments on motor capabilities of children with cerebral palsy. The result from this review will help to inform healthcare practitioners and policymakers on the additional effect of equine-assisted treatments on reducing the burden of cerebral palsy among children.

**Systematic review registration:**

This systematic review protocol is registered with the International Prospective Register of Systematic Reviews (PROSPERO). Registration number: CRD42018096403. This protocol was prepared using the Preferred Reporting Items for Systematic Reviews and Meta-Analyses for Protocols checklist (PRISMA-P).

## Background

Cerebral palsy is one of the major neurological diseases in children showing a prevalence of about 2:1000 live births [1]. It particularly affects children born preterm and necessitates lifelong medical support with physiotherapy being the major therapeutic principle. Equine-assisted treatments of the motor system are increasingly used to improve motor functions in children with cerebral palsy. This includes riding on animal horses but also on so-called artificial horses [2, 3]. Hippotherapy is a distinct form of equine-assisted physiotherapy where certified physiotherapists use the horse as a dynamic tool in a classical physiotherapeutic setting. Further equine-assisted treatments do not necessarily need a certified physiotherapist or therapist but rely on the effects randomly elicited in the rider by the movements of the horse or of the artificial horse.

A large and increasing number of children with cerebral palsy have access to different forms of equine-assisted therapy. As this treatment interacts with a malfunctioning neuromuscular system and aims to improve this pathological medical condition, proof for its therapeutic efficiency is needed. In this context, an increasing number of studies have focused on this question, ranging from single case reports to controlled studies [3, 4]. Outcome measures used in these studies are heterogeneous, including different tests for motor function (for example, the GMFM (Gross Motor Function Measure), pediatric balance scale, SATCo (segmental assessment of trunc control), modified Ashworth scale, quantitative gait analysis) but also electromyographic or comparable measurements [4–6]. A series of reviews has already addressed distinct aspects of these results, frequently only including studies with the GMFM as only outcome measure, none including artificial horses or distinguishing between hippotherapy and further equine-assisted treatments. Therefore, it seems advisable to gain a more global view on the effects of equine-assisted therapies, on the overall effects as well as on differences between distinct treatment modalities.

### Objectives

The aim of this review is to evaluate whether hippotherapy and/or related equine-assisted treatments including artificial horses do influence functional parameters in comparison to usual care in children with cerebral palsy. The proposed systematic review and meta-analyisis will answer the following questions:

- What is the comparative effectiveness of hippotherapy and related equine-assisted treatments compared to standard treatment with regard to motor function (assessed for example by GMFM, electromyographic tests, or movement analysis) in children with cerebral palsy?
- What is the comparative effectiveness of hippotherapy and related equine-assisted treatments compared to standard treatment with regard to quality of life and autonomic function (assessed for example by PEDI (Pediatric Evaluation of Disability Inventory), CHQ (Child Health Questionnaire), Barthel scale) in children with cerebral palsy?
- Is there an advantage in the use of living horses compared to artificial horses?
- Can the observed heterogeneity in the results be explained by study-specific or clinical characteristics?

## Methods

### Eligibility criteria

The selection of studies is based on the following eligibility criteria.

#### Population

We will include pediatric patients with perinatally acquired or congenital cerebral palsy and an age below 18 years. Due to epidemiological reasons in most of these children, cerebral palsy will be related to preterm birth. In a minority, it may be related to alternative reasons such as congenital cerebral malformations or perinatal hypoxia in term-born children.

#### Intervention

We will include trials evaluating animal horse (hippotherapy versus further equine-assisted treatments) or artificial horse therapy (conducted by certified or non-certified therapists) in order to improve motor functions of children with cerebral palsy.

#### Comparator

We will use waitlist controls or non-equine therapies, e.g., physiotherapy or other treatments focusing on the motor system as comparator to horse (animal or artificial)-based therapy.

#### Outcome

We will extract outcomes on motor function, quality of life, and autonomic function. Motor function assessment can be performed by different instruments, e.g., GMFM, electromyographic tests, or movement analysis. Quality of life or performance in daily activities can be assessed, for example, by PEDI (Pediatric Evaluation of Disability Inventory), CHQ (Child Health Questionnaire), or Barthel scale. Autonomic function assessment will be evaluated by, for example, heart rate variability or blood pressure measurements.

#### Study design

We will primarily include parallel-arm or crossover randomized controlled trials (RCTs) and will supplement RCTs with observational cohort studies. Our focus is on hippotherapy or equine-assisted therapy versus any other intervention including “nothing.” Therefore, we will include multi-arm studies if available.

#### Setting

There is no restriction concerning for example distinct standards of treatment or outcome measurement although these data may be included for further analysis.

#### Time frame

There is no restriction concerning for example duration of treatment or duration of follow-up although these data may be included for further analysis.

#### Language and publication status

We will include publications in English, German, French, or Spanish language. We will include studies reported as full text, those published as abstract only, and unpublished data.

### Information sources

We will identify trials through systematic searches of the PubMed, Embase, Web of Science, and Cochrane Central Register of Controlled Trials (CENTRAL) databases using medical subject headings (MeSH) and text words related to hippotherapy and cerebral palsy. Based on the references of the included studies, we will identify further relevant studies. In order to avoid overlooking studies that are ongoing or recently completed, we will also conduct a search of ClinicalTrials.gov (www.ClinicalTrials.gov), the WHO International Clinical Trials Registry Platform (ICTRP) Search Portal (http://apps.who.int/trialsearch/), and the International Prospective Register of Systematic Reviews (PROSPERO) (https://www.crd.york.ac.uk/prospero/) for ongoing or unpublished trials and meta-analyses. In addition, we will contact experts to avoid overlooking relevant information.

### Search strategy

The PubMed search strategy will be developed by the project team. We will perform a preliminary search in the PubMed database in order to identify a first set of publications suitable for inclusion. The reference list of these publications will be searched for further publications to be included. Moreover, from the keywords listed in these publications and by further analyzing these publications, we will identify additional keywords in order to build a final search strategy. This final search strategy will be reused for the PubMed database and will be adapted for the abovementioned further databases. A draft PubMed search strategy is included in the Appendix: A1. Search strategy.

We will search all databases from their inception to the present, and we will restrict our search to English, German, French, or Spanish language of publication or publication status. We will check reference lists of all included studies and any relevant systematic reviews identified for additional references to trials. We will contact authors for missing data and ongoing trials. The searches will be re-run just before the final analyses and further studies retrieved for inclusion.

### Data management

Literature search results will be stored in EndNote. We will identify multiple reports of the same study by matching the order of author names. To avoid double counting, we will include the most embracing report of the particular study only.

### Selection process

The two review authors (MH, NH) will independently assess titles and abstracts of all publications after removing duplicates. Studies which do not meet the inclusion criteria are excluded from further consideration. Possible disagreement will be solved by discussion, and reasons for each excluded study will be recorded. Full texts of the remaining studies will be treated in the same way. The selection process will be documented in a PRISMA flow diagram [7].

### Data collection process

We will use a purposely developed data collection form for study characteristics and outcome data, which has been piloted on at least one study in the review. The two review authors (MH, NH) will independently extract outcome data from included studies to check each others work. We will resolve disagreements by consensus. One review author (NH) will transfer data into the Review Manager (RevMan 5.3.) file. We will double check that data is entered correctly by comparing the data presented in the systematic review to the data collection form.

### Data items

We will extract the following study characteristics:

- Methods: study design, total duration of study, details of any “run in” period, number of study centers and locations, study setting, and date of study
- Participants: *N* randomized, *N* lost to follow-up/withdrawn, *N* analyzed, mean age, age range, gender, severity of condition (e.g., GMFCS (Gross Motor Function Classification System)), inclusion and exclusion criteria, and reported differences between intervention and comparison groups
- Interventions: type of equine-assisted therapy (hippotherapy, not hippotherapy, artificial horse), duration of treatment phase, number of treatments, existence of defined standards of treatment, comparator, concomitant medications or therapies, and excluded medications or therapies
- Outcomes: primary and secondary outcomes specified, and time points reported.
- Notes: funding for trial, and notable conflicts of interest of trial authors.

### Outcomes and prioritization

We will consider motor function assessed by different instruments, e.g., GMFM, electromyographic tests, or movement analysis as primary outcome. Secondary outcomes will address changes related to domains other than motor function, such as functioning during daily life, quality of life (for example, PEDI (Pediatric Evaluation of Disability Inventory), CHQ (Child Health Questionnaire), Barthel scale), or autonomic functions.

### Risk of bias in individual studies

The two review authors (MH, NH) will independently assess risk of bias for each study using the criteria outlined in the revised tool to assess risk of bias in randomized trials (RoB 2.0) or the ROBINS-I tool (Risk Of Bias In Non-randomized Studies - of interventions), respectively (https://sites.google.com/site/riskofbiastool/welcome/rob-2-0-tool). We will resolve any disagreement by discussion. We will assess the risk of bias according to the following domains:

- Bias arising from the randomization process
- Bias in selection of participants into the study
- Bias due to confounding
- Bias in classification of interventions
- Bias due to deviation from intended interventions
- Bias due to missing outcome data
- Bias in measurement of the outcome
- Bias in selection of the reported result

We will grade each potential source of bias as low risk, some concerns, or high risk and provide a quote from the study report together with a justification for our general judgment as low, moderate, serious, or critical risk of bias. We will summarize the risk of bias judgments across different studies for each of the domains listed.

### Data synthesis

We will undertake meta-analyses only where this is meaningful, i.e., if the treatments, participants, and the underlying clinical question are similar enough for pooling to make sense. Otherwise, we will summarize our findings in structured tables and interpret them narratively.

In parallel group trials, we do not expect unit of analysis issues to occur. In simple two treatment-two period crossover trials, patients undergo both treatments. Thus, the number of observations does not match the number of units that were randomized. To take the unit of analysis issue in crossover trials into account, we will estimate the treatment effect as average crossover difference. Given the clinical heterogeneity across trials on cerebral palsy patients and their differences in co-morbidities and co-medications, we will use random-effects meta-analysis to produce an overall summary of average treatment effect across trials. We will present results as the pooled meta-analytic effect estimate with corresponding 95% confidence interval, and the estimates of the between study variability *τ*^2^, and *I*^2^ to describe the percentage of the variability in effect estimates that is due to heterogeneity. We will analyze dichotomous data as risk ratios (RR) with 95% confidence intervals (CI). For continuous data, we will use the mean difference with 95% CI for outcomes measured in the same way between trials. We will use the standardized mean difference (SMD) with 95% CI to combine data of the primary outcome motor function, which are expected to be measured by using different instruments. We will enter data presented as a scale with a consistent direction of effect. We will visually inspect the forest plots to assess the degree of heterogeneity, in addition to measuring it with the *I*^2^ statistic and *Q* test for heterogeneity. If we identify substantial heterogeneity, we will investigate it using subgroup analyses and sensitivity analyses. We will regard heterogeneity as substantial if (a) the *I*^2^ value is high (exceeding 50%) and (b) there is inconsistency between trials in the direction or magnitude of effects (judged visually), or there is a low *p* value (≤0.10) in the *Q* test for heterogeneity.

We plan to carry out the following subgroup analyses:

- Type of equine-assisted therapy: hippotherapy versus no hippotherapy horse-riding
- Type of horse: therapy with living horses versus therapy with artificial horses
- Duration of therapy

Where subgroup analyses are performed, we will restrict them to the primary outcomes. In addition, we will use the formal test for subgroup interaction in RevMan. We will perform sensitivity analysis by limiting analyses to studies at low risk or some concerns of bias. Moreover, we will explore the impact of using different analysis models to evaluate the robustness of the results. This will be done by fitting a fixed-effect model to the data of the primary outcome.

### Meta-bias

For all included studies, we will check if a registered study protocol is available and whether the protocol was registered before the study was initialized. Moreover, we will screen the outcomes documented in the protocol against the reported outcomes to evaluate potential reporting bias. If we are able to pool more than 10 trials, we will create and examine a funnel plot to explore possible publication bias for the primary outcome by assessing funnel plot asymmetry visually and by using Egger’s test at a significance level of 5%. If there are fewer than 10 studies included in the meta-analysis, we will assess publication bias qualitatively based on the characteristics of the included studies.

### Confidence in cumulative evidence

We will judge the confidence of the achieved results using the GRADE (Grading of Recommendations Assessment, Development and Evaluation) approach [8].

## Conclusion

This is the first systematic review on equine-assisted treatments which aims to differentiate between the effects of true hippotherapy which always requires a certified and specifically trained physiotherapist to conduct treatment and further equine-assisted treatments. It is also the first systematic review which aims at comparing the effects of animal horses to the effects of artificial or “robo” horses. Depending on the data quality, it will be possible to establish conclusive results with regard to all these questions, to reach partial results, or to recommend which kind of studies should be conducted in future time in order to answer these questions. Major difficulties of the intended review may arise if the outcome data are based on heterogeneous outcome measures other than the GMFM which can actually be considered the gold standard. One further difficulty may occur if non-satisfactory control data are provided and/or if data are based on case reports or small case series only.

## Appendix: A1. Search strategy

Example search strategy for PubMed: (https://www.ncbi.nlm.nih.gov/PubMed/):

(((“cerebral palsy”[MeSH Terms] OR (“cerebral”[All Fields] AND “palsy”[All Fields]) OR (“cerebral palsy”[All Fields])) AND (“hippo”[All Fields] AND (“therapy”[All Fields]))) OR ((“therapeutics”[All Fields] OR “therapeutic”[All Fields]) AND (“horses”[MeSH Terms])) OR ((“horses”[All Fields] OR“horse”[All Fields] OR “equidae”[MeSH Terms] OR “equidae”[All Fields] OR “equine-assisted”[MeSH Terms] OR “equine-assisted”[All Fields]) AND “therapy”[MeSH Terms]) OR (“equine-assisted therapy”[All Fields]) OR (“equine”[All Fields] AND “assisted”[All Fields] AND “therapy”[All Fields)

## Data Availability

Data sharing is not applicable to this article as no datasets were generated or analyzed during the current study.

## Abbreviations

CENTRAL: Cochrane Central Register of Controlled Trials
CHQ: Child Health Questionnaire
CI: Confidence interval
GMFM: Gross Motor Function Measure
GRADE: Grading of Recommendations Assessment, Development and Evaluation
ICTRP: International Clinical Trials Registry Platform
MeSH: Medical subject headings
PEDI: Pediatric Evaluation of Disability Inventory
PROSPERO: Prospective Register of Systematic Reviews
RCT: Randomized controlled trial
ROBINS-I: Risk Of Bias In Non-randomized Studies - of interventions
RR: Risk ratio
SATCo: Segmental assessment of trunc control
SMD: Standard mean difference
WHO: World Health Organization
